# The effects of virtual reality technology on negative emotions in the elderly: a meta-analysis

**DOI:** 10.3389/fpsyg.2025.1636780

**Published:** 2025-10-07

**Authors:** Miao Liu, Weisi Zeng, Surong Liu

**Affiliations:** School of Nursing, Chengdu University of Traditional Chinese Medicine, Chengdu, Sichuan, China

**Keywords:** virtual reality, virtual reality technology, older adults, negative emotions, meta-analysis

## Abstract

**Objectives:**

We explored whether older individuals’ negative emotions were modified via virtual reality technology.

**Methods:**

We conducted computer searches of four Chinese databases (CNKI, Wanfang, VIP, CBM) and four English databases (Embase, PubMed, Web of Science, Cochrane Library) from inception to February 12, 2025. Two researchers independently screened titles, abstracts, and full texts according to predefined inclusion and exclusion criteria, resolving discrepancies through discussion. The PRISMA 2020 flow diagram summarizes the study selection process. Meta-analysis was performed using RevMan 5.4 software.

**Results:**

A total of 14 studies were included. The combined MD (95% CI) values and *p*-values were as follows: anxiety (SMD = −0.63; 95% CI: −0.82 to −0.45; *P*<0.05), depression (SMD = −0.49; 95% CI: −0.79 to −0.20; *P*<0.05), geriatric depression (WMD = −1.44; 95% CI: −2.57 to −0.31; *P*<0.05), and sleep quality (WMD = −1.94; 95% CI: −3.05 to −0.84; *P*<0.05). Fear of falling (WMD = −0.32; 95% CI: −2.81 to 2.16; *p* > 0.05) was not statistically significant, whereas the remaining outcomes all showed significant differences.

**Conclusion:**

While virtual reality technology showed no significant effect on fear of falling, it appeared to improve depression, anxiety, and sleep quality in older adults. However, due to heterogeneity among studies, further high-quality studies are required to confirm these findings.

**Clinical trial registration:**

https://www.crd.york.ac.uk/prospero/display, ID=CRD42024623259.

## 1 Introduction

The proportion of the global population aged 60 years or older has been rising rapidly in recent years. According to the World Health Organization (2025), the average global life expectancy was projected to reach 73.3 years in 2024. The number of people worldwide aged 60 years and older is predicted to rise from roughly 1.1 billion in 2023 to 1.4 billion by 2030, and then to 2.1 billion by 2050. The health of elderly people is receiving an increasing amount of focus as the implications of population aging gradually permeate. The public’s physical health functions weaken as they age, their social horizons are limited, and they are more likely to experience pain, mental health issues and emotional anguish. Positive and negative emotions constitute the two dimensions that form emotions. An assortment of unpleasant or painful emotional experiences can be referred to as negative emotions. These emotions are part of the human emotional experience and often include fear, sadness, depression, and anxiety (Ma and Zhang, 2022). With regard to an individual’s mental health and quality of life, both positive and negative emotions serve as important indicators of wellbeing. Emotions are also directly related to mortality risk in the elderly, which may be increased by negative emotions. According to estimates from the World Health Organization (2023), mental disorders affect 14% of individuals aged 60 years and older globally, accounting for 10.6% of the disability burden in this age group. Depression is estimated to affect 5.7% of older adults worldwide.

Anxiety and depression are the most prominent mental health challenges that affect one in four people who are elderly, according to the World Health Organization’s Global Burden of Disease research (de la Casa-Marín et al., 2024). One of the most prevalent mental illnesses in the elderly is depression, and as humanity ages, their physiological capacity goes down and they are made more predisposed to both acute and chronic illnesses. From a neurological standpoint, the decline of neural processes and structural brain aging may be partially a cause of the rising incidence of mood disorders in the elderly. According to a study, people with anxiety and depression had “brain ages” that were around 2.8–2.9 years older than their real ages (Han et al., 2021). This implies that these kinds of mental disorders could be related to neurodegenerative changes in the elderly. In addition, a recent analysis pointed out that neuroinflammation weakens the emotional and cognitive regulating systems by inhibiting hippocampus neurogenesis and neuroplasticity. Higher levels of chronic inflammation in the aging brain may thus decrease resilience, rendering older adults more prone to depression and cognitive decline. This effect may be especially apparent when combined with other aging-related risk factors (Tian et al., 2025). Multiple chronic inflammation markers, such as suPAR, were found to be significantly correlated with social isolation and loneliness in multi-cohort studies. These findings suggest that social isolation could harm the psychological and physiological resilience of older adults by encouraging “inflammatory aging,” which boosts the risk of depression, cognitive decline, and other conditions (Matthews et al., 2024). One study discovered that by raising disease-related stressors (such as pain and physical functional limits), chronic illnesses or multiple comorbidities directly increase the burden of depressive symptoms in seniors. Through both sociopsychological mediation and physiological-functional impairment, chronic diseases worsen older persons’ psychological vulnerability (Mindlis et al., 2023). The distribution of mental disorders across life stages is highly unequal, according to global estimates from the Global Burden of Disease (GBD) study. A significant portion of prevalence and years lived with disability (YLD) are tied to childhood and adolescence, especially for those aged 10 to 24. Moreover, the overall prevalence of mental disorders in older adults, based on diagnostic criteria, is often lower than that of younger age groups (GBD 2019 Mental Disorders Collaborators, 2022). The overall prevalence of psychological disorders among the 5–24 age group is 11.63%, which is much higher than in older age groups, in line with Kieling’s age-specific analysis based on GBD data (Kieling et al., 2024). Although epidemiological data indicate that younger populations generally have higher prevalence rates of mental disorders than older adults, the World Health Organization reports that the global suicide rate among older adults is approximately 27.2% (World Health Organization, 2023). It has additionally been shown that the costs of healthcare for patients with depression are 1.86 times higher than those for patients lacking depression, and that the growing elderly population, coupled with associated psychological issues, presents significant obstacles to the community (Primavera et al., 2024).

Pharmacological and non-pharmacological psychological therapies are currently utilized to treat psychological disorders in the elderly. Targeted techniques must be used to treat multiple psychological issues. In recent years, non-pharmacological therapies have become the preferred treatment for mental disorders in older adults, as long-term use of pharmacological interventions may have adverse effects, such as accelerating cognitive decline, thereby jeopardizing their health (Reallon et al., 2024). Physical exercise, daily debate, psychoeducation, music therapy, cognitive and behavioral therapy, acceptance and commitment therapy, and reminiscence therapy only comprise a few of the specific approaches which qualify under the broad category of non-pharmacological therapies (Sun et al., 2022). At the time, mindfulness techniques are also growing in demand; nonetheless, beginners find it difficult to focus for extended periods of time (Cinalioglu et al., 2023). There is an urgent need for new types of interventions for dealing with psychosocial problems in older adults, given that psychological interventions are often exorbitant, while there are fewer specialists available, which renders them unobtainable for the average family. Furthermore, medication side effects can make it difficult for frail older adults to maintain their heath over time. Interventions for psychological disorders in the elderly should ideally be long-lasting, individualized, safe, and free of adverse effects. However, non-pharmacological interventions are constrained by time, financial resources, and adherence, whereas pharmacological treatments are frequently impacted by multimorbidity and side effects in real-world situations. Alternatives such as music therapy, fitness therapies, or telepsycho-education have been tested by researchers to close this gap, but the outcomes have been mixed. In this context, virtual reality (VR) is gradually being recognized as a potential alternative that more closely approximates ideal treatment conditions.

As an emerging technology, virtual reality (VR) may provide users with dynamic, interactive and multi-sensory virtual experiences (Doniger et al., 2018). VR technology offers a controlled, multi-sensory immersive experience by integrating users’ visual and auditory senses within a virtual environment (Cho et al., 2024). VR technology is commonly classified into three categories: fully immersive, semi-immersive, and non-immersive (Szczepocka et al., 2024). The degree of perception of the real world and the sensation of immersion in the virtual world can be adjusted through various VR devices. The most prevalent type of virtual reality is non-immersive, in which a user interacts with the virtual world utilizing a computer, tablet, or smartphone while maintaining a sense of their outside world. Semi-immersive VR typically incorporates screens, haptic feedback devices, infrared cameras, and other devices that allow users to interact with the virtual world while remaining aware of the physical environment. In order to obtain full visual coverage, immersive virtual reality usually makes use of input devices such as hand controllers and head-mounted displays (Salatino et al., 2023). VR systems have been proposed as an ideal platform for cognitive and motor training programs as they can gather user task data, send feedback as required and be integrated with electronics that include motion capture, eye tracking, and health monitors. In addition to creating advanced virtual settings, virtual reality technology also incorporates motor and cognitive training that boosts users’ motivation for continuous involvement (Kwan et al., 2021). Further, the field of mental health has increasingly utilized virtual reality technologies. The results of Restout’s study (2025), which assessed the effect of a virtual reality intervention on the mental health of elderly residents of nursing homes, reported a significant improvement in residents’ mental health, demonstrating the potential of VR as a tool for mental health interventions in this setting. Taken together, recent randomized controlled trials and meta-analyses have shown that virtual reality can reduce anxiety (Yang et al., 2025) and depression (Huang and Yang, 2022), improve quality of life and social engagement (Wang, 2024), and lessen older adults’ fear of falling (Saragih et al., 2025). Nevertheless, some of these effects diminished once the intervention was suspended, suggesting that the sustainability of intervention effects requires further clarification in future applications and research.

The effectiveness of VR technology in interventions for older adults is well-recognized. However, several challenges must be addressed for its benefits to be fully realized. Reviews have shown that the main barriers to the promotion of VR in elderly and long-term care settings include funding and maintenance costs, insufficient infrastructure such as networks and venues, and staff training and workload constraints, resulting in a situation described as “willingness to adopt but difficulty in normalization” (Hung et al., 2023). This suggests that it is difficult to implement interventions on a large scale without stable budgets, appropriate facilities, and staffing support. In a nursing home study, older people’s acceptance of VR was strongly associated with previous experience of technology and the availability of on-site carer assistance; the study also suggested that equipment costs and affordability affect engagement and sustained use (Li et al., 2024). Another systematic review highlighted that older adults engaging in immersive VR may experience motion-related symptoms or discomfort. It is recommended that future research should increase the sample size and quality of randomized controlled trials, optimize the dosage and duration of use, and improve comfort and ease of interaction (Drazich et al., 2023b). In addition to such practical barriers, regulatory and policy frameworks may restrict the use of VR in healthcare or nursing institutions. Manufacturers and clinical organizations must comply with regulatory standards (such as FDA requirements for medical devices and EU MDR requirements for medical software) and establish procedures for risk management and adverse event reporting if VR is classified as a medical device or medical software. At the financial level, healthcare insurance or institutional funding rules in some countries may limit the allocation of funds for costly and complex technologies, thereby constraining the adoption of VR in hospitals and nursing homes (U.S. Food and Drug Administration, 2022; Centers for Medicare and Medicaid Services (CMS), 2023; European Commission — Directorate-General for Health and Food Safety, 2024). Therefore, in order to provide practical recommendations for institutional adoption, both device compliance and financial feasibility should be considered when evaluating clinical evidence for the effects of VR on mood in older adults.

Recent meta-analyses indicate that the number of VR interventions targeting negative emotions in older adults remains limited and highly heterogeneous. For instance, a meta-analysis of randomized controlled trials (Yang et al., 2025) found that VR significantly improved quality of life and reduced anxiety and depression in older adults with chronic illnesses. However, the evidence was limited by variations in intervention forms, immersion levels, and outcome measures. A review of VR reminiscence therapy (Mao et al., 2024) reported similarly inconsistent findings. While benefits were observed for anxiety and memory, the evidence for depression, apathy, and quality of life was weak, and some studies even reported functional decline at 3–6 months of follow-up. Most previous systematic reviews included fewer than 20 randomized controlled trials, and differences in study designs, outcome measures, and follow-up duration further undermined the reliability of the findings. In the present study, applying strict inclusion and exclusion criteria, only 14 eligible trials were included, underscoring the scarcity of high-quality, homogeneous research in this field and providing a foundation for future standardized studies. Therefore, a systematic and rigorous quantitative synthesis is urgently needed to clarify the true efficacy of VR interventions for negative emotions in older adults. This study aims to address this gap by evaluating the impact of virtual reality technology on negative emotions in older adults, with the ultimate goal of informing its clinical application in mental health.

## 2 Methods

### 2.1 Search strategy

This study was conducted in accordance with the Preferred Reporting Items for Systematic Reviews and Meta-Analyses (PRISMA 2020) (Page et al., 2021) to develop the PICOS search principles: P (older people aged 60 years and over), I (VR interventions), C (routine care), O (depression, anxiety, sleep quality, etc.), and S (RCTs). Four Chinese databases (CNKI, VIP, CBM, and WanFang Data) and four English databases (The Cochrane Library, PubMed, Embase and Web of Science) were searched, and the literature that met the inclusion criteria was reviewed. The search period spanned from the establishment of the database to 12 February 2025. We used the same core keywords (“virtual reality,” “elderly,” “negative emotions,” and “randomized controlled trials”) across all databases and adjusted the search terms appropriately based on the characteristics of each database (e.g., PubMed used MeSH terms, Embase used Emtree, and Chinese databases such as CNKI used Chinese subject terms). A combination of subject terms and free-text search strategies was employed, supplemented by manual literature screening. The complete search strategies for each database are provided in Supplementary Table 1.

### 2.2 Inclusion and exclusion criteria

#### 2.2.1 Inclusion criteria

(a) Study subjects: aged ≥60 years, race and gender are not limited; (b) Type of study: randomized controlled trial (RCT). This study only included individual or cluster RCTs and did not include quasi-experimental designs or non-randomized controlled studies; (c) Intervention: virtual reality interventions for the experimental group, and conventional care or medication for the control group; (d) Outcome indicators: Geriatric Depression Scale (GDS), Hamilton Anxiety Rating Scale (HAMA), Hamilton Depression Rating Scale (HAMD), Pittsburgh Sleep Quality Index (PSQI), International Fall Efficacy Scale (FES-I), etc.; at least one of these scales must have been reported; (e) Only literature published in English and Chinese was included.

#### 2.2.2 Exclusion criteria

(a) Literature without full-text access; (b) Dissertations, conference papers, systematic evaluations, etc.; (c) Studies with data that could not be converted, or with important data are missing. For missing or incomplete data, we attempted to contact the original authors for additional information. If this was not possible, the study was excluded; (d) Duplicate publications of the same study.

This study was registered in the PROSPERO (CRD42024623259).

### 2.3 Literature screening and data extraction

**Figure 1:**
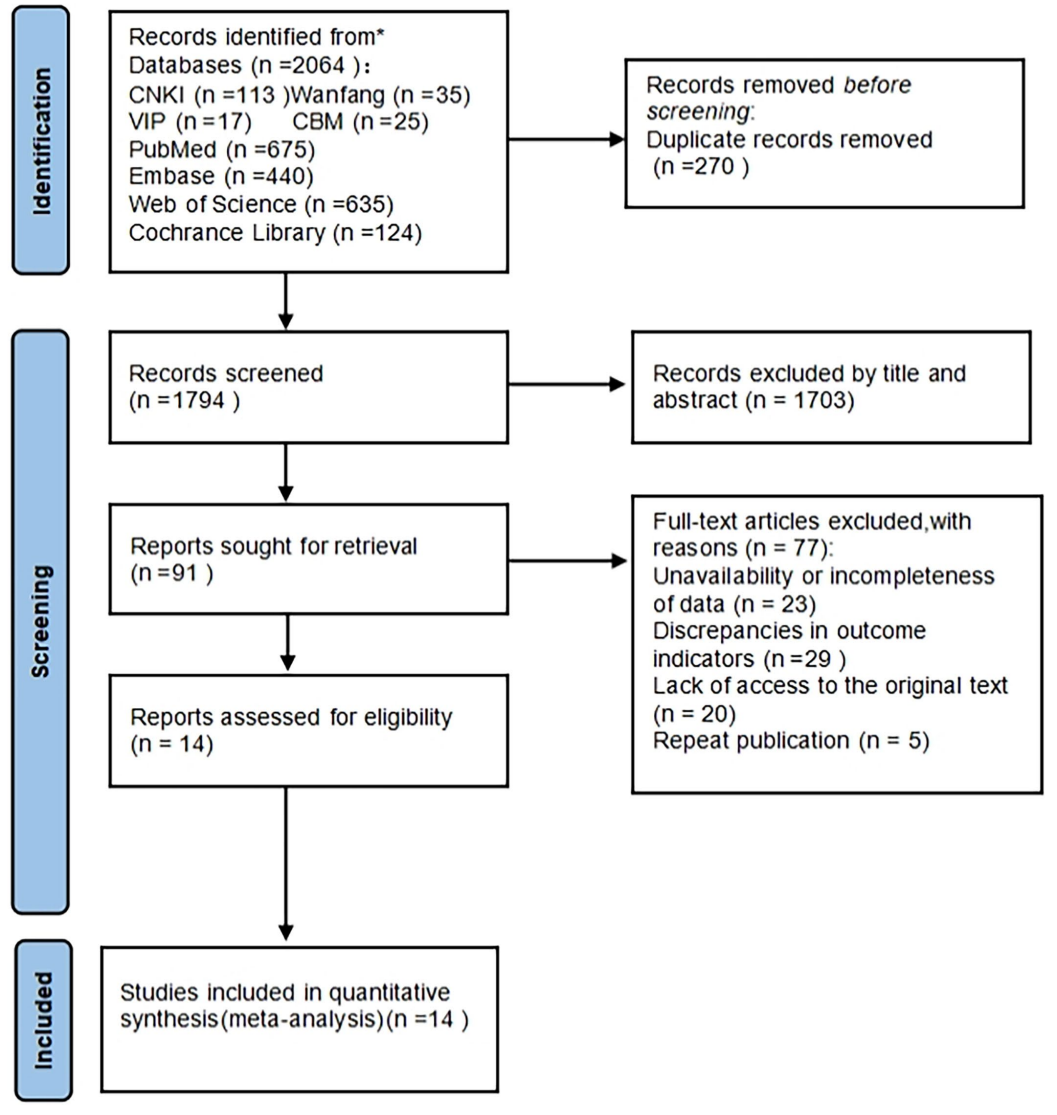
Literature screening flow chart.

The literature screening process followed the PRISMA protocol, including removal of duplicate documents, title and abstract screening, full-text evaluation, and final inclusion of studies. The specific process is shown in Figure 1. Firstly, according to the inclusion and exclusion criteria, all the literature retrieved and imported into EndNote X9 software was preliminarily screened, and duplicates and obviously unsuitable literature were removed by reading the titles and abstracts, and then the rest of the literature was downloaded and read in full text, and according to the content of the literature and the criteria of the exclusion criteria, the literature that needed to be included was determined eventually. Literature screening was done independently by two researchers with evidence-based training, and the results were cross-checked. If disagreement was encountered, a third researcher was consulted to reach a consensus result.

Data extraction was performed independently by two researchers using a pre-designed Excel template, which included fields for basic study information (author, year, country), study design, sample characteristics, intervention measures, control conditions, outcome measures, and primary results. Since this tool is based on graded judgments rather than specific numerical assessments, the *κ* value was not calculated in this study; however, all assessments ultimately reached consensus. The specific assessment results for each study are presented in Supplementary Table 2. Data were entered into Excel for management and organization, and basic information to be included in the literature was extracted: title of the literature, first author, time of publication, country of publication, type of study, total sample size, characteristics of the study population, mode and duration of intervention, outcome indicators, and duration of follow-up.

### 2.4 Quality evaluation

Quality assessment of the included studies was conducted independently by two researchers using the Literature Quality Evaluation Tool (LQET) from the Cochrane Handbook for Systematic Reviews (Higgins et al., 2011). The evaluation covered: random sequence generation, allocation concealment, blinding of the implementation process, bias in outcome measurement, completeness of outcome data, selective reporting, and other biases. Entries were judged as follows: “low risk” as grade A, some “unclear” but not “high risk” as grade B, and some “high risk” as grade C. Only studies graded A or B were finally included. Any disagreements were resolved through discussion with a third researcher. Since this tool is based on graded judgments rather than specific numerical assessments, this study did not calculate the *κ* value, but ultimately, all assessments were consistent. The specific assessment results of each study are shown in Supplementary Table 2.

### 2.5 Statistical analysis methods

The data from each trial were meta-analyzed in this study using the Review Manager (RevMan) 5.4 software from the Cochrane Collaboration. The standardized mean difference (SMD) and its 95% confidence interval were used for continuous variables if the scales or assessment instruments used in various studies were inconsistent; if the scales were consistent, the weighted mean difference (WMD, output as MD in Rev. Man) and its 95% confidence interval were used. At *p* < 0.05, a difference was deemed statistically significant. The I^2^ test was used to measure heterogeneity. A fixed-effect model was applied when I^2^ < 50% and *p* > 0.10; a random-effects model was applied when I^2^ ≥ 50% or *p* < 0.1. Subgroup analyses were planned to explore sources of heterogeneity when the number of studies and available data permitted; sensitivity analyses (excluding individual studies one by one) were also conducted to assess the robustness of the results. Publication bias was assessed using a funnel plot when ≥10 studies were included; if the number of studies was insufficient, the Egger’s test or Begg’s test in Stata 14.0 software was considered as a Supplementary material.

## 3 Results

### 3.1 Literature search and inclusion results

A total of 2,064 records were initially retrieved. After removing duplicates, 1,794 records remained and were screened by title and abstract. Ninety-one full-text articles were assessed for eligibility. Ultimately, 14 studies met the inclusion and exclusion criteria and were included in the meta-analysis. The detailed process of study selection is presented in Figure 1 (PRISMA flowchart).

### 3.2 Basic characteristics of the literature

Fourteen papers were finally included in the study, and the countries studied were mainly China, including seven papers, followed by Brazil and Poland with two papers each. Two in Chinese (Shi et al., 2023; Wang et al., 2020), 12 in English (Anguera et al., 2017; Cheng et al., 2020; Cieslik et al., 2023; Fan et al., 2022; Gomes et al., 2018; Monteiro-Junior et al., 2017; Montero-Alía et al., 2019; Qiu et al., 2024; Stanmore et al., 2019; Szczepańska-Gieracha et al., 2021; Wan et al., 2024.; Wong et al., 2024). Fourteen were randomized controlled studies involving 1778 older adults. The characteristics of the included literature are detailed in Table 1.

**Table 1:** Basic characteristics of the included literature.

Included literature	Time	Country	Sample size N (T/C)	Intervention		Intervention time	Conclusion indicator
				(Control group)	(Experimental group)		
Shi, Y. Y.	2023	China	58/51	Conventional drugs	Fully immersive VR scene	6 days	②③④
Wang, L.	2020	China	108/108	Conventional drugs	Fully immersive VR scene Game strength training combined with biofeedback techniques	12 weeks	②③④
Anguera, J. A.	2017	USA	10/12	Routine cognitive training	Non-immersive VR therapeutic video games	8 weeks	③
Cheng, V, Y	2020	China	24/24	Routine care	Fully immersive VR scenarios, interactive games combined with aromatherapy	9 weeks	②
Cieslik, B.	2023	Poland	30/30	Routine care	Fully immersive VR garden scenarios	4 weeks	①③④
Fan, C. C.	2022	China	30/32	Routine activities	Fully immersive VR scenarios combined with horticultural activities	8 weeks	①
Gomes, G. C. V.	2018	Brazil	15/15	Routine care	Non-immersive VR interaction with games based on Wii system	7 weeks	①⑤
Monteiro-Junior, R. S.	2017	Brazil	9/9	Exercise	Fully immersive VR scenario-based exercise game	3 weeks	⑤
Montero-Alía, P.	2019	Spain	274/356	Routine care	Non-immersive VR game interaction based on Wii system	12 weeks	⑤
Qiu, T.	2024	China	100/100	Traditional Tai Chi	Fully immersive VR Tai Chi Chuan	24 weeks	①
Stanmore, E. K	2019	UK	50/56	Routine care	Non-immersive VR video sports games	12 weeks	①⑤
Szczepańska-Gieracha, J.	2021	Poland	12/11	Routine care	Fully immersive VR garden scenes	4 weeks	①③④
Wan, Y.	2024	China	32/20	Conventional medicine	Total immersion VR scene with breathing training	6 weeks	②③④
Wong, A. K. C.	2024	China	101/101	Conventional care	Total immersion VR scene game	18 weeks	①

T, Trial group; C, Control group; ① GDS, ② PSQI, ③ HAMD or HADS-D, ④ HAMA or HADS-A, ⑤ FES-I.

### 3.3 Results of literature quality evalution

**Figure 2:**
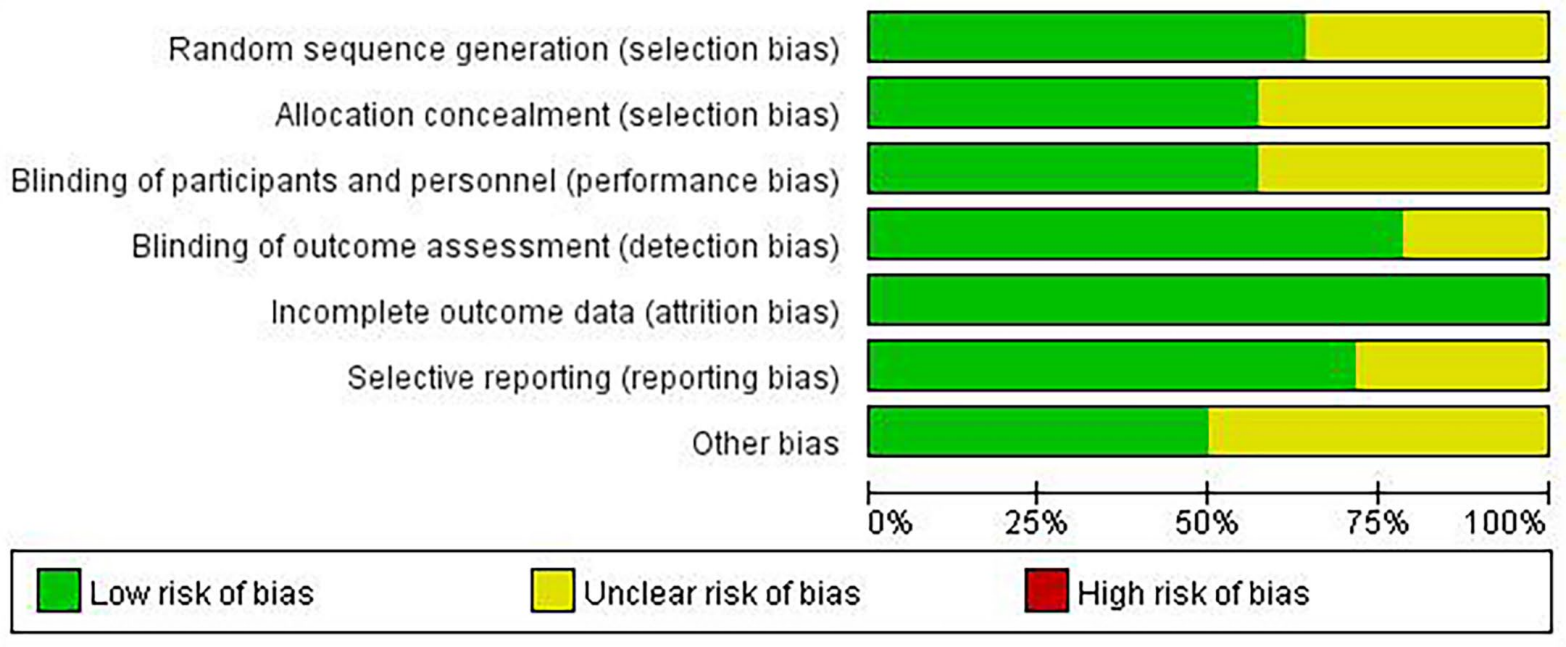
Literature quality evaluation chart.

According to the Cochrane Collaboration’s quality evaluation of the 14 included studies, two (Shi et al., 2023; Montero-Alía et al., 2019) were rated as grade A, and 12 (Anguera et al., 2017; Cheng et al., 2020; Cieslik et al., 2023; Fan et al., 2022; Gomes et al., 2018; Monteiro-Junior et al., 2017; Qiu et al., 2024; Stanmore et al., 2019; Szczepańska-Gieracha et al., 2021; Wang et al., 2020; Wan et al., 2024; Wong et al., 2024) were rated as grade B. Five studies were rated as “unclear” for random sequence generation, six for allocation concealment, six for blinding of implementation, and three for outcome measurement bias. Fourteen studies were rated as “low risk” for completeness of outcome data, and four as “unclear” for reporting outcomes. Seven studies were rated as “unclear” for other biases. Further details are provided in Figure 2 and Supplementary Table 2.

### 3.4 Meta-descriptive analysis

#### 3.4.1 Geriatric depression scores

**Figure 3:**
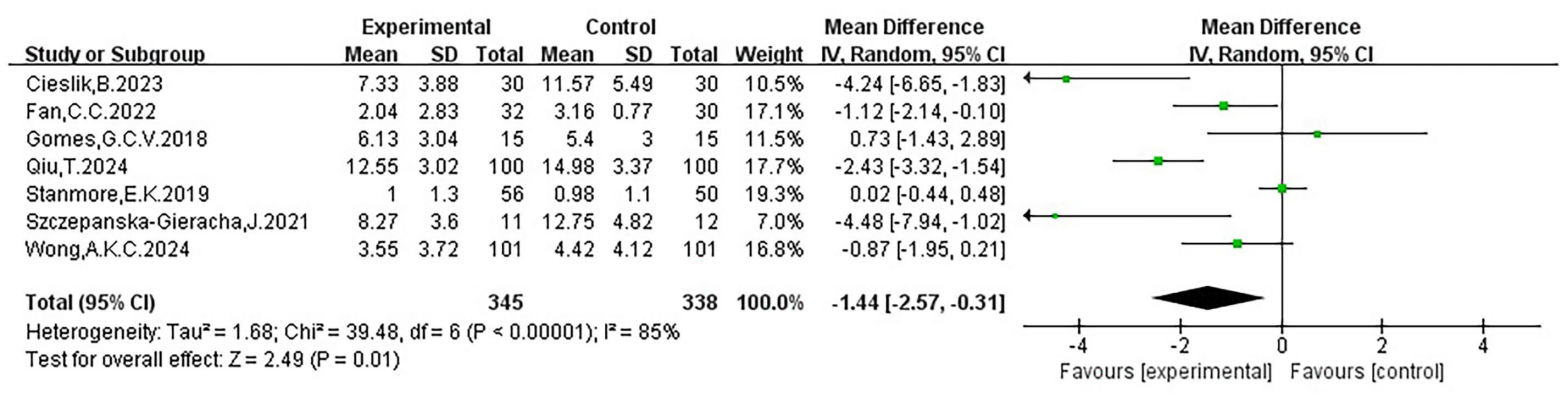
Forest plot of geriatric depression.

The seven included studies (Cieslik et al., 2023; Fan et al., 2022; Gomes et al., 2018; Qiu et al., 2024; Stanmore et al., 2019; Szczepańska-Gieracha et al., 2021; Wong et al., 2024) indicated that most effect sizes were positioned to the left of the null line, suggesting that VR interventions generally helped alleviate depressive symptoms in the elderly. However, three studies reported wide confidence intervals (CIs), reflecting uncertainty due to limited sample sizes or differences in study designs. Additionally, three studies reported CIs crossing the zero line, indicating inconsistent results. Nevertheless, the overall pooled effect supported a beneficial role of VR in alleviating depression in older adults (WMD = −1.44, 95% CI: −2.57 to −0.31, *p* < 0.05). Substantial heterogeneity was observed (I^2^ = 85%), suggesting significant variability in study results, potentially due to differences in population characteristics or intervention methods. The mean values, standard deviations, sample sizes, and results of each study are presented in Figure 3 and Table 2.

**Table 2:** Meta-analysis results.

Conclusion indicator	Inclusion of studies	Number of patient cases	Heterogeneity test		effect model	Meta-analysis effect value	
			Ι^2^	*P*		WMD or SMD (95%CI)	*P*
Anxiety	5	460	1	0.40	Fixed	−0.63(−0.82, −0.45)	<0.00001
Depression	6	482	50	0.08	Random	−0.49(−0.79, −0.20)	0.0009
Geriatric depression	7	683	85	<0.00001	Random	−1.44(−2.57, −0.31)	0.01
Sleep quality	4	425	80	0.002	Random	−1.94(−3.05, −0.84)	0.0006
Fear of falling	4	784	80	0.002	Random	−0.32(−2.81, 2.16)	0.80

#### 3.4.2 Depression scores

**Figure 4:**
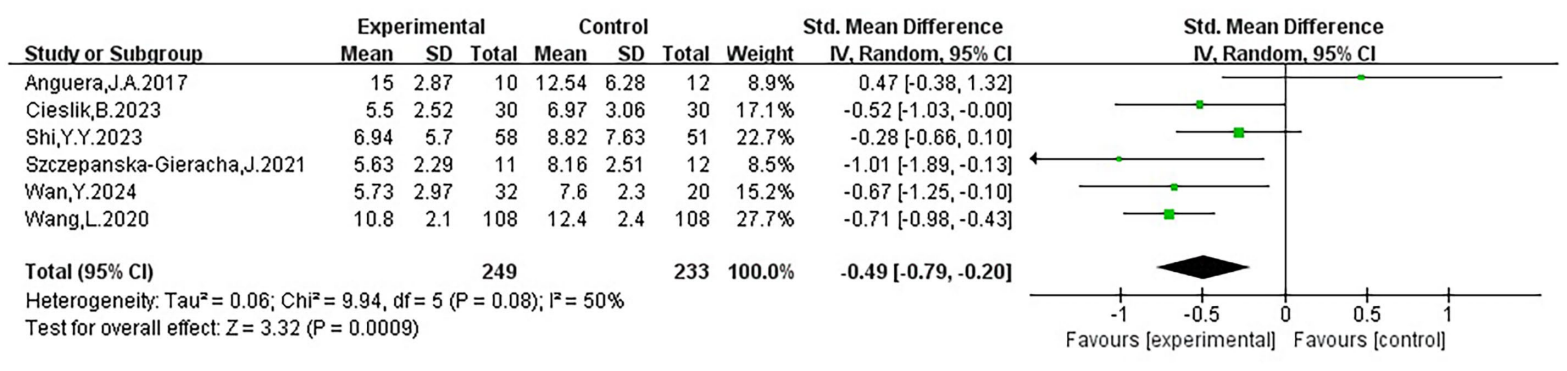
Forest plot of depression.

Six studies (Shi et al., 2023; Wang et al., 2020; Anguera et al., 2017; Cieslik et al., 2023; Szczepańska-Gieracha et al., 2021; Wan et al., 2024) indicated that virtual reality interventions had a significant positive effect on depression (SMD = −0.49, 95% CI: −0.79 to −0.20, *p* < 0.05), with moderate heterogeneity (I^2^ = 50%), suggesting some differences across studies, possibly related to variations in sample size or assessment tools. The forest plot showed that five studies lay to the left of the no effect, while one study lay to the right. In terms of confidence intervals (CIs), two studies reported relatively narrow intervals, whereas four had wider intervals, suggesting limited stability in some findings; among these, two studies’ CIs crossed the line of no effect, and one study’s CI just touched it, indicating inconsistency in individual results. Nevertheless, the pooled effect indicated a favorable impact of VR interventions on depression symptoms. Detailed data and findings from each study are presented in Figure 4 and Table 2.

#### 3.4.3 Anxiety scores

**Figure 5:**
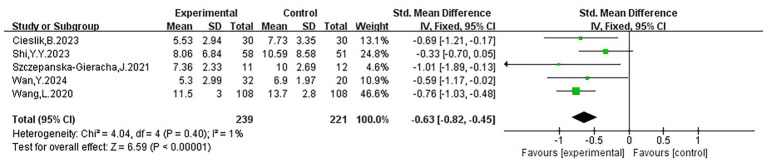
Forest plot of anxiety.

The meta-analysis results of five studies (Shi et al., 2023; Wang et al., 2020; Cieslik et al., 2023; Szczepańska-Gieracha et al., 2021; Wan et al., 2024) demonstrated that VR interventions significantly reduced anxiety symptoms in older adults (SMD = −0.63, 95% CI: −0.82 to −0.45, *p* < 0.00001), with no evidence of heterogeneity (I^2^ = 1%). The forest plot showed that the effect sizes of all studies lay to the left of the line of no effect, indicating consistent directionality. Three studies reported moderately narrow confidence intervals, while two presented wider intervals, suggesting weaker stability in some findings; one study’s CI crossed the line of no effect. Nevertheless, the pooled effect size robustly supported the beneficial impact of VR interventions on anxiety. Detailed data and results of each study are provided in Figure 5 and Table 2.

#### 3.4.4 Sleep quality scores

**Figure 6:**
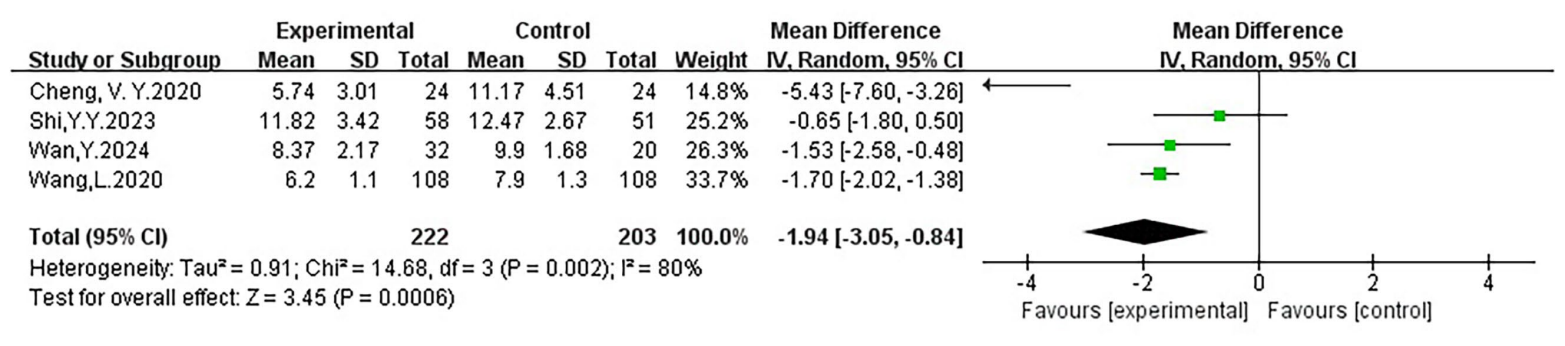
Forest plot of sleep quality.

Four studies (Wang et al., 2020; Cheng et al., 2020; Wan et al., 2024; Shi et al., 2023). Evaluated the effects of VR on sleep quality. The pooled analysis showed that VR significantly improved sleep quality in older adults (WMD = −1.94, 95% CI: −3.05 to −0.84, *p* < 0.05), although heterogeneity was high (I^2^ = 80%), possibly due to differences in sample sizes and intervention strategies. The forest plot indicated that all effect sizes were on the left of the lines of no effect, suggesting a consistent direction of benefit. Most studies had moderate CI widths, one study had a narrower CI, and one study’s CI crossed the lines of no effect, reflecting some uncertainty. Overall, the evidence supports the beneficial role of VR in improving the quality of sleep. Figure 6 and Table 2 display the particular data and findings from each study.

#### 3.4.5 Fear of falling scores

**Figure 7:**
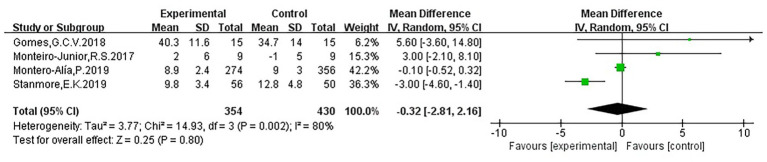
Forest plot of fear of falling.

Four studies (Gomes et al., 2018; Monteiro-Junior et al., 2017; Montero-Alía et al., 2019; Stanmore et al., 2019) examined the effects of VR on fear of falling. The pooled results showed no statistically significant effect (WMD = −0.32, 95% CI: −2.81 to 2.16, *p* > 0.05), with high heterogeneity (I^2^ = 80%), possibly due to differences in participant characteristics and intervention duration. The forest plot indicated that two studies reported effect sizes to the left of the line of no effect and two to the right, reflecting inconsistent findings. One study had a narrow CI, one had a moderate CI, and two had wide CIs, with three studies’ CIs crossing the line of no effect, suggesting considerable uncertainty. Overall, the current evidence is insufficient to confirm a significant impact of VR on reducing fear of falling in older adults. Each study’s particular data and findings are presented in Table 2 and Figure 7.

### 3.5 Sensitivity analysis

Separately using the two effects model analysis and then comparing the results, the results show that the MD value, 95% CI and *p* value of the random effects model combined with the fixed effects model did not change much, indicating that the meta-analysis results are more stable. A sensitivity analysis was conducted by sequentially excluding individual studies. For depression, the overall results remained stable, and most exclusions did not materially change the pooled effect. However, when Cieslik et al., 2023 and Qiu et al. (2024) were excluded, the effect size decreased, the 95% CI crossed zero, and the statistical significance was attenuated, indicating that these studies influenced the overall estimate. When Wang et al. (2020) was excluded, the pooled SMD decreased to −0.41 (95% CI: −0.77 to −0.05, *p* = 0.02), remaining statistically significant but closer to the significance threshold. Excluding the remaining studies produced negligible changes. Overall, the findings for depression appear robust, with a small number of studies exerting greater influence on the pooled estimate. For sleep quality, sequential exclusion of individual studies did not materially alter the overall conclusions, indicating stable results. The pooled effect was slightly reduced when Cheng et al. (2020) was removed, suggesting this study strengthened the overall effect, while the exclusion of Shi et al. (2023) and Wan et al. (2024) marginally increased the pooled effect. Removing Wang et al. (2020) attenuated the statistical significance (WMD = −2.31, 95% CI: −4.44 to −0.19, *p* = 0.03), implying some influence of this study on the robustness of the findings. For fear of falling, the leave-one-out analysis showed no meaningful changes in effect size or statistical significance. Although the effect direction shifted when Montero-Alía et al. (2019) and Stanmore et al. (2019) was removed, neither result reached significance, confirming that VR has no consistent effect on this outcome. For anxiety, no sensitivity analysis was undertaken given the minimal heterogeneity (I^2^ = 1%). Full details of the sensitivity analyses are presented in Supplementary Table 3.

### 3.6 Publication bias analysis

It was not examined for publication bias utilizing the funnel plot, given that there were only seven publications included in this study for the elderly depression outcome indicator, which failed to meet the threshold of ≥10 publications for analysis. We used Stata 14.0 to conduct Egger’s tests for outcomes with ≥3 studies. No evidence of publication bias was observed for sleep quality (*t* = 0.21, *p* = 0.851), anxiety (*t* = −0.24, *p* = 0.829), depression (*t* = 0.66, *p* = 0.545), elderly depression (*t* = − 0.16, *p* = 0.879), or fear of falling (*t* = 0.11, *p* = 0.926). Nevertheless, as all outcomes included fewer than 10 studies, the statistical power of these tests was limited.

## 4 Discussion

### 4.1 Meta-analysis result

One of the most prevalent mental health issues affecting older adults is anxiety, which has a significant negative impact on their quality of life. According to Andreescu et al. (2011), older adults are more likely to develop anxiety disorders, as they are less likely than younger people to use psychological terminology to describe their emotions, minimize symptoms, or attribute them to physical illnesses. In addition, they are less able to recognize and address anxiety promptly. From a physiological perspective, studies have reported that VR interventions combined with relaxation music significantly reduce serum cortisol and adrenocorticotropic hormone (ACTH) levels, indicating a potential role in regulating stress-related hormonal responses. In addition, the immersive nature of VR can redirect attention away from anxiety-inducing stimuli, foster a sense of safety and relaxation, and thereby help alleviate anxiety symptoms (Singh et al., 2024). In line with the findings of the current meta-analysis, VR-based therapies show statistically significant effects in reducing anxiety symptoms among older adults. These findings are consistent with those of Niki et al. (2021), who reported no adverse effects. This may be because VR reminiscence therapy immerses patients in an immersive virtual environment, which helps them recall past experiences, reduces stress, and promotes multisensory mindfulness-based healing. The results also concur with those of Appel et al. (2020), showing that VR interventions can reduce anxiety levels in older adults with physical disabilities living in residential care facilities. This may be attributed to the fact that the virtual environment offered by VR can transcend the spatial limitations of the physical environment and allow users to experience the beauty of the virtual world, thereby enhancing positive emotional experiences and fostering inner peace. Our findings indicate that VR interventions can alleviate anxiety in older adults; however, variability across studies warrants attention. For example, Restout et al. (2025) observed that although VR improved depression and quality of life in nursing home residents, it did not significantly reduce anxiety. In contrast, Yang et al. (2025) reported significant reductions in anxiety among older adults with chronic illnesses. Such discrepancies may be attributable to contextual factors, including living environment, baseline psychological characteristics, intervention frequency, and content. These findings underscore the need for future research to better delineate target populations and intervention conditions. The five studies included in this meta-analysis were all randomized controlled trials, and the interventions in the experimental groups were all fully immersive virtual reality environments. The results seem to suggest that the immersive experience delivered by VR may account for its anxiolytic effect. “Presence,” or the feeling of being in virtual reality, is the subjective term for the immersive experience. The Place Illusion (PI) and the Plausibility Illusion (Psi) are the two concepts that constitute Presence (Freeman et al., 2017). The idea behind PI is that we use our whole body to create the impression of being in a virtual place. Psi is the feeling that virtual reality events are happening, even when one is aware that they are not. Participants may act more authentically in VR when both PI and Psi are active. The greatest benefit of virtual reality is that people are aware that the environment is not real, but their bodies and minds behave as though it is. As a result, they are more likely to encounter challenging circumstances in virtual reality than in real life, and may engage with novel therapeutic approaches before applying what they have learned to the real world. Virtual reality technology may therefore be considered a preferred treatment modality, as it can help patients receive more effective psychotherapy.

According to the findings of this meta-analysis, VR exerts a statistically significant impact on depression symptoms in older adults. The results corroborate those of Kim et al. (2023) and suggest that middle-aged women may benefit from VR’s antidepressant effects. Potential explanations include that VR, when combined with exercise-based games in which users receive instant feedback, experience enjoyment, spark curiosity, and increase participation, may alleviate depressive symptoms. In addition, an enjoyable gaming experience that motivates users to engage in physical activity can further reduce such symptoms. The outcomes are in line with those of Drazich et al. (2023a,b), further indicate that older adults in urban areas benefit from VR-based physical activity. It is possible that VR-based physical activity is more entertaining and engaging for users, thereby improving treatment adherence. It may also provide older adults with a more comfortable and secure setting that encourages continued participation. Therefore, we think that, by using virtual worlds to activate reward-related brain circuits, VR can successfully reduce depression. According to a brain network model of late-life depression, these reward systems interact with dysfunction in the prefrontal—default mode—executive control network to contribute to the persistence of depression (Gunning et al., 2021). Furthermore, studies have demonstrated that, in positive emotion or reward tasks, activation of key regions of the reward system (such as the nucleus accumbens and ventral striatum) is significantly reduced in depressed patients (Solomonov et al., 2023). Even though the majority of research points to VR’s ability to help older adults with depression, not all studies are in agreement. Another study compared VR-CBT with conventional interventions, and while both showed alleviating effects on depressive symptoms, there were no significant differences between the groups, suggesting that VR is not always superior to traditional treatments (Lee et al., 2024). For instance, a subgroup analysis revealed that VR intervention was not significantly effective in individuals over 60 who had suffered a stroke, possibly because sensory decline affected the VR user experience (Liu et al., 2023). On the whole, all studies utilized psychometric scales to assess the impact of the intervention on depressive symptoms, with evidence supporting the validity and reliability of the instruments used (Pocklington, 2017). Even so, there was a high degree of heterogeneity in the results. Hospital-specific measures, which include the Hospital Anxiety and Depression Scale, have been used in certain research, while measures tailored to older persons, notably the Geriatric Depression Scale, have been used in others. All of the tests measure depression, yet they may measure distinct types of depression. It is also possible that the interventions covered a wide range of periods, and this may have shaped the results of the studies that included depressive outcomes. These research investigations also involved multiple countries, and each country offered a different measure of VR, ranging from fully immersive experiential scenarios to incorporating other elements of exercise training. The creation of established treatment protocols and long-term assessment of intervention effects could help to maximize the effectiveness of VR in older adults with psychological issues and encourage its broader adoption, as there are currently no standardized treatment procedures for VR therapies.

This meta-analysis demonstrated that VR significantly enhanced older adults’ sleep quality. The results are consistent with the findings of Yan et al. (2024), who observed that preoperative VR use improved the sleep quality of cardiac surgery patients, highlighting the considerable potential of VR in the non-pharmacological treatment of sleep disorders. This may be attributable to the fact that VR offers a multimodal experience, facilitates psychological pre-adaptation, minimizes fear of the unknown, and promotes better sleep. Furthermore, Xu et al. (2022) reported that VR combined with cognitive therapy was more effective in treating adolescent insomnia. This may be because traditional cognitive behavioral therapy involves a lengthy treatment period and poor patient compliance, whereas pharmacological treatment can lead to drug dependence and memory problems. While its advantages are not always apparent, our research suggests that VR may improve older adults’ sleep quality. Differences may exist between subjective experiences and physiological sleep architecture. For example, one study found that although VR meditation increased subjective sleep ratings among intensive care unit patients, objective sleep duration remained unchanged (Kim and Kang, 2025). Similarly, another study reported that although VR gaming generated positive emotions and modulated the autonomic nervous system, it did not significantly improve sleep metrics (Rubio-Arias et al., 2024). These contradictory results highlight the need for more rigorous study designs and longer follow-up periods in future research to determine VR’s true effectiveness across different elderly populations. Regarding the mechanisms, diminished sleep quality in the elderly partly stems from a pronounced reduction in nocturnal melatonin peaks, despite minimal overall diurnal variation. This suggests that disrupted circadian melatonin release may impair sleep initiation and maintenance (Scholtens et al., 2016). Moreover, by reducing blue light interference, promoting mental and physical relaxation, and stimulating parasympathetic nervous system activity, the immersive environment of virtual reality may enhance sleep stability and regulate endogenous sleep patterns. VR interventions have been shown to significantly increase parasympathetic activity while reducing sympathetic arousal, thereby improving autonomic nervous system balance in older adults with chronic insomnia, which in turn contributes to better sleep quality and mental wellbeing (Wan et al., 2024). Alternatively, VR can circumvent time and space constraints, effectively improve patient compliance, and have undetectable side effects, all of which fully illustrate the wide range of potential applications for VR in patients with sleep disorders. An objective and reasonably priced way of enhancing mental stability is through meditation. Virtual reality-based meditation has been shown to have considerable practical utility in enhancing sleep quality, lowering stress levels, and regulating the autonomic nervous system in nursing students in early adulthood (Kim et al., 2024). In this study, realistic natural visuals were used to enhance focus and promote mental stability, and this technique yielded beneficial outcomes. The physical environment, including light, sound, and visual input, has long had a significant impact on sleep. Recent research suggests that hypnotic sounds may improve sleep quality (Riedy et al., 2021), and listening to calming music can influence blood pressure, heart rate, and respiration, all of which affect sleep. Music therapy therefore continues to serve as a complementary approach to sleep therapy (Huang et al., 2022). Moreover, visual stimulation from light can also influence sleep; Tang et al. (2021) examined how audio-visual stimulation affected both sleep quality and musculoskeletal discomfort in older adults. The aforementioned indicates that VR may be the reason older adults’ sleep quality has improved. It delivers audio-visual stimulation and establishes the impression that the user is in a completely different setting, which affects physiological changes in the body and strengthens the quality of sleep in a comfortable setting. The gender and age variations among the enrolled respondents may be the cause of the significant degree of variability in the sleep quality study’s results. Some study participants were exclusively female, whereas other groups had a higher percentage of male participants. Gender may affect the outcome of the intervention due to biological differences and the ideological conceptions that men and women hold differently as a result of society, culture, etc. Future investigations should be carried out since some of the study participants were younger than 60, and some were older than 80. The physiological changes brought on by age-related changes and co-morbidities of the organism may result in inconsistent results. Future research should examine how age-related physiological changes and co-morbidities interact, as these factors may also produce conflicting outcomes.

Based on the conclusions of this meta-analysis, older people’s fear of falling is not statistically affected by virtual reality and its effectiveness in reducing this fear remains inconclusive. According to the findings of Mohamed Zein El-AbdeenMohamed et al. (2025), VR combined with rehabilitation activities may assist frail older adults who are afraid of falling. Many commercial virtual gaming systems can be integrated with VR technology to detect changes in the real world and user movements. This enables prompt feedback, which can enhance motivation and encourage continued participation. Exercising at home through VR may help older adults improve their physical capacity and reduce their fear of falling. VR-based physical activity has thus emerged as a feasible option for maintaining the health of older people, offering a wide range of at-home exercise opportunities. Consistent with the results of a 6-week randomized controlled trial, VR balance training improved balance and reduced fear of falling among nursing home residents (Zahedian-Nasab et al., 2021). Such training may strengthen muscle, stepping ability, balance, and mood, thereby benefitting older adults at risk of falling. Moreover, visual feedback during simulation exercises may increase participants’ self-efficacy and awareness of balance control. Engaging in structured VR-based activities, such as guided workouts, may also improve confidence in balance and alleviate fear of falling. Nevertheless, the overall usefulness of VR in this regard remains debated. While Chen et al. (2024) concluded in a systematic review that VR is not effective in lowering fear of falling, another systematic review by Gao et al. (2024) reported that VR therapy improved gait, balance, and lower-limb strength in older adults without disabilities, thereby reducing fall risk. In summary, although some studies suggest that VR interventions may hold potential for reducing fear of falling in older adults, others have failed to observe significant improvements, indicating that the evidence in this area remains inconsistent. Based on the findings of this review, considerable uncertainty persists regarding the efficacy of VR for this outcome. Future research, therefore, requires more high-quality, multicenter, randomized controlled trials with long-term follow-up to further validate and clarify its role. The results of this study should be interpreted with caution, as fear of falling is influenced by multiple factors, including sociodemographic characteristics, physical function, fall history, balance, underlying medical conditions, and psychological or cognitive status. Moreover, the duration of interventions and follow-up varied substantially across studies, and some had no follow-up at all, which may have reduced the statistical power of short-term effects. The meta-analysis also revealed substantial heterogeneity (I^2^ = 80%), which may be attributed to the relatively small number of included studies and their geographical dispersion across countries with different environmental and healthcare contexts. This heterogeneity may have diluted the overall effect, leading to pooled findings that failed to reach statistical significance. Future studies should therefore aim to establish standardized VR intervention protocols for older adults, including clear definitions of training frequency, duration, and difficulty progression. In addition, larger sample sizes and multicenter collaborations are needed to enhance generalizability. To improve comparability and robustness of results, consistent use of validated fall fear assessment scales and longer follow-up periods is essential. Furthermore, the integration of wearable technology or big data monitoring with intelligent VR programs may provide more objective evaluations and support the development of personalized fall prevention strategies.

### 4.2 Strengths and limitations

Due to the fact that VR equipment enables users to participate in fun, game-like activities while getting immediate feedback, many reviews portray VR as an engaging and motivating tool. Virtual reality (VR) is being used in recent research for rehabilitation (Chen et al., 2022). As of right now, VR equipment in task-oriented practice can be employed as a medical device. Another plus of VR rehabilitation is that it incorporates repetitive, task-oriented exercises which are customized to each person’s skill level for at-home workouts. In light of their small size, safety, lack of external forces, and ease of management, virtual reality systems hold a lot of potential for home rehabilitation (Ase et al., 2025). However, the majority of research findings only partially report outcomes and often lack accurate, objective, and positive data. Most VR software and systems also do not have suitable evaluation criteria. In addition, VR equipment remains expensive and has not been widely implemented across diverse populations. Three-dimensional virtual environments still require improvements in realism, and related technologies remain limited due to challenges in development and optimization. Some VR systems are still in their infancy, and the use of VR equipment can cause adverse effects in certain users, including motion sickness, visual fatigue, ocular discomfort, balance and spatial orientation difficulties, and head or neck discomfort. Consequently, it is essential to monitor users’ responses during use, implement necessary modifications, and promptly address any concerns.

Furthermore, although VR has demonstrated positive outcomes under experimental conditions, its feasibility in real-world settings, such as homes or care facilities, requires further validation. A comprehensive assessment of factors that may affect the scalability and utility of VR interventions—such as equipment costs, accessibility, staff training, and optimal intervention duration—is typically lacking in existing studies.

### 4.3 Implications for practice

Most of the current studies are in the preliminary stage; in order to verify the effect of virtual reality technology on negative emotions such as anxiety and depression in the elderly, more studies are still needed. First, in accordance with research evidence, countries should swiftly introduce and learn from virtual reality (VR), bolster technology research and development, and apply it. In subsequent studies, researchers should appropriately broaden the scope of the application object to explore more areas and continue to optimize the procedure so as to minimize the negative reactions of users in the intervention. Meanwhile, it is crucial to reserve the adaptation time of the VR equipment when conducting the study design. At last, VR should be combined with traditional intervention methods as reinforcement and demonstration. Second, while employing virtual reality technology, we should first determine the sort of intervention program which must be implemented for the intervention population and encourage a more personalized and concentrated intervention program. Following that, we should standardize the intervention process, starting with establishing the user’s motion sickness condition and steadily increasing tolerance in terms of intervention time and content. Ideally, the user’s intervention process ought to involve psychological scales and physiological indicators, such as heart-rate monitoring and eye-tracking. It is desirable to have psychological scale assessment and physiological indicator monitoring, such as heart and eye tracking, throughout the user intervention process. Last but not least, various departments should work together more and devote more effort to the research and development of virtual reality technology. Humanistic care should be integrated into VR research and development, along with the process’s use to supply people with true VR services. R&D institutions ought to boost their collaboration with hospitals, assisted living facilities, and other organizations.

## 5 Conclusion

It is recommended that more randomized controlled studies with rigorous design and long-term follow-up be conducted in future studies to further explore the effect of virtual reality technology on the role of negative emotions in older adults and to improve the effectiveness of the clinical treatment of psychosocial problems in older adults. Virtual reality technology has significantly improved anxiety, depression, and sleep quality in older adults, yet it is uncertain whether it has improved their fear of falling.

## Data Availability

The original contributions presented in the study are included in the article/Supplementary material. For further inquiries, please contact the corresponding author/s.
