# Supplementary material for: The effects of virtual reality technology on negative emotions in the elderly: a meta-analysis
Source: Front Psychol. 2025 Oct 7;16:1636780. doi: 10.3389/fpsyg.2025.1636780 (PMC12550953; doi:10.3389/fpsyg.2025.1636780)
Supplement: Supplementary file 1 [file Table_1.docx]

**Supplementary Materials Table 1 Search strategies for each database**

| Database | Search terms and search strategies | Notes |
| --- | --- | --- |
| PubMed | ("virtual reality"[MeSH] OR "virtual environment"[Title/Abstract] OR "video game"  [Title/Abstract] OR "virtual reality technology"[Title/Abstract] OR"VR"[Title/Abstract] OR "gaming"[Title/Abstract] OR "exergaming"[Title/Abstract]) AND ("Aged"[MeSH] OR "elderly"[Title/Abstract] OR "elderly people"[Title/Abstract] OR "old people"  [Title/Abstract] OR "elder"[Title/Abstract] OR "aged people"[Title/Abstract]) AND  ("Depression"[MeSH] OR "Anxiety"[MeSH] OR "negative emotion*") AND  ("Randomized Contronlled Trial"[Publication Type] OR "controlled clinical trial"  [Title/Abstract] OR "randomized"[Title/Abstract] OR "randomized controlled  experiment"[Title/Abstract] OR "a randomised controlled study"[Title/Abstract] OR  "RCT"[Title/Abstract]). | Used MeSH + free-text, restricted to RCTs |
| Embase | ('virtual reality'/exp OR 'virtual reality':ti,ab) AND ('aged'/exp OR 'elderly':ti,ab) AND ('depression'/exp OR 'anxiety'/exp OR 'negative emotion*') AND ('randomized controlled trial'/exp OR 'randomized':ti,ab) | Used Emtree terms + free-text, restricted to RCTs |
| Web of Science | TS=("virtual reality") AND TS=("older adults" OR "elderly") AND TS=("depression" OR "anxiety" OR "negative emotions") AND TS=("randomized controlled trial" OR "RCT") | Used “Topic” search field, limited to RCTs |
| Cochrane Library | ("virtual reality" OR "VR") AND ("older adults" OR "elderly") AND ("depression" OR "anxiety" OR "negative emotions") AND ("randomized controlled trial" OR "RCT") | Use keyword free word combinations to search for intervention studies and systematic reviews |
| CNKI | ("虚拟现实" OR "虚拟环境" OR "虚拟游戏" OR "虚拟康复" OR "情景互动" OR "VR") AND ("老年人" OR "老人") AND ("抑郁" OR "焦虑" OR "负性情绪") AND ("随机对照" OR "随机对照试验") | Chinese subject terms + free-text |
| Wanfang | ("虚拟现实" OR "虚拟环境" OR "虚拟游戏" OR "虚拟康复" OR "情景互动" OR "VR") AND ("老年人" OR "老人") AND ("抑郁" OR "焦虑" OR "负性情绪") AND ("随机对照" OR "随机对照试验") | Similar to CNKI |
| VIP | ("虚拟现实" OR "虚拟环境" OR "虚拟游戏" OR "虚拟康复" OR "情景互动" OR "VR") AND ("老年人" OR "老人") AND ("抑郁" OR "焦虑" OR "负性情绪") AND ("随机对照" OR "随机对照试验") | Similar to CNKI |
| CBM | ("虚拟现实" OR "虚拟环境" OR "虚拟游戏" OR "虚拟康复" OR "情景互动" OR "VR") AND ("老年人" OR "老人") AND ("抑郁" OR "焦虑" OR "负性情绪") AND ("随机对照" OR "随机对照试验") | Similar to CNKI |
