# Supplementary material for: The effects of virtual reality technology on negative emotions in the elderly: a meta-analysis
Source: Front Psychol. 2025 Oct 7;16:1636780. doi: 10.3389/fpsyg.2025.1636780 (PMC12550953; doi:10.3389/fpsyg.2025.1636780)
Supplement: Supplementary file 2 [file Table_2.docx]

| First Author(Year) | Sample Size n (T/C) | LQET Rating |
| --- | --- | --- |
| Shi, Y. Y.2023 | 58/51 | A |
| Wang, L. 2020 | 108/108 | B |
| Anguera,J.A.2017 | 10/12 | B |
| Cheng,V, Y2020 | 24/24 | B |
| Cieslik,B.2023 | 30/30 | B |
| Fan,C.C.2022 | 30/32 | B |
| Gomes,G.C.V.2018 | 15/15 | B |
| Monteiro-Junior, R.S.2017 | 9/9 | B |
| Montero-Alía, P.2019 | 274/356 | A |
| Qiu,T.2024 | 100/100 | B |
| Stanmore,E.K2019 | 50/56 | B |
| Szczepańska-Gieracha,J.2021 | 12/11 | B |
| Wan,Y.2024 | 32/20 | B |
| Wong,A.K.C.2024 | 101/101 | B |

**Supplementary Materials Table 2 Literature quality evaluation**
