# Supplementary material for: The effects of virtual reality technology on negative emotions in the elderly: a meta-analysis
Source: Front Psychol. 2025 Oct 7;16:1636780. doi: 10.3389/fpsyg.2025.1636780 (PMC12550953; doi:10.3389/fpsyg.2025.1636780)
Supplement: Supplementary file 3 [file Table_3.docx]

**Supplementary Material Table 3 Results of sensitivity analysis**

| Exclusion studies | WMD | 95%CI | P | Conclusions Changes |
| --- | --- | --- | --- | --- |
| Do not eliminate  (senile depression) | -1.44 | -2.57，-0.31 | < 0.05 | With statistical significance |
| Eliminate Cieslik,B.2023 | -1.09 | -2.19，0.01 | 0.05 | Weaken |
| EliminateFan,C.C.2022 | -1.56 | -2.94，-0.17 | 0.03 | Stable |
| Eliminate Gomes,G.C.V.2018 | -1.73 | -2.95，-0.51 | 0.006 | Stable |
| Eliminate Qiu,T.2024 | -0.87 | -1.95，0.21 | 0.04 | Weaken |
| Eliminate Stanmore,E.K.2019 | -1.75 | -2.89，-0.62 | 0.003 | Stable |
| Eliminate Szczepanska-Gieracha,J.  2021 | -1.20 | -2.33，-0.07 | 0.04 | Stable |
| Eliminate Wong,A.K.C.2024 | -1.60 | -2.98，-0.22 | 0.02 | Stable |
| Exclusion studies | SMD | 95%CI | P | Conclusions Changes |
| Not to eliminate (depression) | -0.49 | -0.79,-0.20 | 0.0009 | With statistical significance |
| Eliminate Anguera,J.A.2017 | -0.58 | -0.78，-0.38 | ＜0.00001 | Stable |
| Eliminate Cieslik,B.2023 | -0.48 | -0.84，-0.12 | 0.009 | Stable |
| Eliminate Shi,Y.Y.2023 | -0.55 | -0.90，-0.21 | 0.002 | Stable |
| Eliminate Szczepanska-Gieracha,J.  2021 | -0.44 | -0.75，-0.14 | 0.005 | Stable |
| Eliminate Wan,Y.2024 | -0.45 | -0.80，-0.10 | 0.01 | Stable |
| Eliminate Wang,L.2020 | -0.41 | -0.77，-0.05 | 0.02 | Stable |
| Exclusion studies | WMD | 95%CI | P | Conclusions Changes |
| Not to eliminate  (sleep quality) | -1.94 | -3.05，-0.84 | 0.0006 | With statistical significance |
| Eliminate  Cheng,V, Y2020 | -1.49 | -2.02，-0.95 | ＜0.00001 | Stable |
| Eliminate Shi,Y.Y.2023 | -2.45 | -3.87，-1.03 | 0.0007 | Stable |
| Eliminate Wan,Y.2024 | -2.25 | -3.95，-0.56 | 0.009 | Stable |
| Eliminate Wang,L.2020 | -2.31 | -4.44，-0.19 | 0.03 | Stable |
| Exclusion studies | WMD | 95%CI | P | Conclusions Changes |
| Not to eliminate  (Fear of falling) | -0.32 | -2.81,2.16 | 0.80 | No statistical significance |
| Eliminate Gomes,G.C.V.2018 | -0.72 | -3.27,1.82 | 0.58 | Stable |
| Eliminate Monteiro-Junior,R.S.2017 | -0.91 | -3.66,1.84 | 0.52 | Stable |
| Eliminate Montero-Alía,P.2019 | 0.77 | -4.70,6.25 | 0.78 | Stable |
| Eliminate Stanmore,E.K.2019 | 0.89 | -1.68,3.45 | 0.50 | Stable |
